# A Large-Scale Replication of the Effectiveness of the KiVa Antibullying Program: a Randomized Controlled Trial in the Netherlands

**DOI:** 10.1007/s11121-020-01116-4

**Published:** 2020-05-12

**Authors:** Gijs Huitsing, Gerine M. A. Lodder, Wiliam J. Browne, Beau Oldenburg, Rozemarijn Van der Ploeg, René Veenstra

**Affiliations:** 1 Department of Sociology, University of Groningen, and Interuniversity Center for Social Science Theory and Methodology (ICS), Groningen, The Netherlands; 2 Department of Developmental Psychology, Tilburg University, Tilburg, The Netherlands; 3 School of Education, University of Bristol, Bristol, UK; 4 Department of Pedagogy and Educational Science, University of Groningen, Groningen, The Netherlands

**Keywords:** Bullying, Evidence-based intervention, KiVa, Prevention, Randomized controlled trial, Replication, Victimization

## Abstract

This study evaluates the effectiveness of the KiVa antibullying program in the Netherlands through a randomized controlled trial of students in grades 3–4 (Dutch grades 5–6). The sample involved 98 schools who volunteered to participate in the research, with 245 classes and 4383 students at the baseline (49% girls; *M* age = 8.7 years), who participated in five measurement waves, collected in three consecutive school years. After the baseline, two-thirds of the schools were assigned to the intervention condition (KiVa or KiVa+, the latter included an additional intervention component of network feedback to teachers) and one-third to the control condition (waiting list, care as usual) with a stratified randomization procedure. The effects of the intervention on self-reported victimization and bullying were tested using cross-classified ordered multinomial models and binomial logistic regression models. These longitudinal models showed that self-reported victimization and bullying reduced more strongly in KiVa-schools compared with control schools, with stronger effects after two school years than after one school year of implementation. The results showed that for students in control schools, the odds of being a victim were 1.29–1.63 higher, and the odds of being a bully were 1.19–1.66 higher than for KiVa students. No significant differences between KiVa and KiVa+ emerged. Overall, the findings provide evidence of the effectiveness of the KiVa program in the Netherlands.

**Electronic supplementary material:**

The online version of this article (10.1007/s11121-020-01116-4) contains supplementary material, which is available to authorized users.

Bullying is a common problem in primary and secondary schools and exacts high costs to society (McDaid et al. 2017). Victims are at increased risk of severe negative short- and long-term consequences, such as poor mental (depression, anxiety, and lower self-esteem) and physical health (see for an overview Arseneault 2018), as well as unemployment, lower income, and poverty (Brimblecombe et al. 2018). Long-term negative consequences have also been documented for bullies, who are at increased risk of becoming involved in delinquency, crime, and alcohol abuse (Ttofi et al. 2011). These negative consequences evidently demonstrate the need for effective antibullying intervention programs. The most effective antibullying interventions are, on average, able to reduce bullying by 20% (Gaffney et al. 2018). These programs focus on the school context in which bullying occurs. The school-wide approach takes bullying as a systemic problem, involving individual, classroom, and school level causes.

The KiVa antibullying program is such a school-wide approach. This study aimed to replicate the effectiveness-study of the KiVa antibullying program (Kärnä et al. 2011b; Salmivalli et al. 2010) in the Netherlands. Replication research is important to test if the intervention overcomes the challenges associated with cultural and contextual adaptation and implementation in a different context, such as using materials in another educational setting, gaining support for the intervention, or adjusting organizational structures (Nocentini and Menesini 2016; Sundell et al. 2014).

## KiVa Antibullying Program

KiVa—the Finnish word for “nice” and an acronym for “against bullying”—aims to enable students and teachers to tackle bullying (Salmivalli et al. 2010). KiVa builds on the rationale that bullying is often the result of a group process, and that group members can also stop it. For that reason, it is important that schools target not only bullies or victims but also the group as a whole (Salmivalli 2010).

Three insights provide support for the rationale of the group and the group’s teacher in preventing bullying. First, the participant role approach (Salmivalli et al. 1996) suggests that all children are involved in bullying in some way, even if they do not bully themselves. Bystanders of bullying can assist or reinforce bullies, ignore the situation, or defend victims. Second, bullies often have a strong position in the peer group, are seen as popular, and set the bullying norm in the class (Dijkstra et al. 2008; Volk et al. 2012). If relevant others in their peer group intervene against bullies or encourage others to stop, bullying becomes less attractive. KiVa addresses the participant roles and high status of bullies by enabling bystanders to show that they are against bullying, to make them support the victim, and to no longer encourage the bully. Third, teachers can be role models to prevent bullying (Saarento et al. 2015; Veenstra et al. 2014). If teachers stand up against bullying, it becomes easier to make everyone in the classroom responsible for creating a climate in which bullying is rejected. Emphasizing the teachers’ role is important, given that they are often unaware of bullying incidents and do not always intervene appropriately (Yoon et al. 2014). Therefore, teachers were trained to teach children safe strategies to support victims and to change group norms in such a way that bullying behavior becomes associated with low status.

The KiVa materials have two aims, to prevent future bullying and victimization, and to intervene in cases where bullying emerges (Salmivalli et al. 2010). Prevention involves universal theme-lessons targeted at all students by teachers, and indicated actions targeted at ongoing bullying by specialized KiVa team members, which are directed at students who have been identified as victims or bullies (see Appendix 1 for all KiVa components). Teachers, principals, and school counselors participated in an interactive 2-day START-training, delivered by professional school trainers, prior to implementing the intervention.

The KiVa program has been evaluated in Finland in large randomized controlled trials (Kärnä et al. 2011b for grades 4–6; 2013 for grades 1–3 and 7–9) and in a nationwide rollout (Kärnä et al. 2011a). The findings from the randomized controlled trial (RCT) for grades 4–6 materials (used in the current study) showed that both victimization and bullying were significantly reduced (Kärnä et al. 2011b): the odds of being a victim or bully were about 1.3 times and 1.5 times higher, respectively, for control school students than for KiVa school students. Moreover, in grades 1–3, the effects were more pronounced for girls, and there were small effects favoring KiVa in grades 7–9. KiVa was also effective in an RCT in Italy, but with stronger effects in grade 4 (ORs for victimization and bullying 1.93 and 1.31) than grade 6 (ORs 1.21 and 1.33, Nocentini and Menesini 2016).

## The Present Study

In 2011, the success of KiVa in Finland and the lack of an evidence-based antibullying program in the Netherlands led to funding by the Dutch government to investigate the effectiveness of KiVa in the Netherlands, by a program called Onderwijs Bewijs (Evidence-Based Education). This program funded experimental designs to educational interventions. We implemented the core components of KiVa that are expected to produce the intended reductions of bullying and victimization (Herkama and Salmivalli 2013): teacher training, theme lessons, virtual learning environment, KiVa-symbols such as recess vests and posters, parent materials, and indicated actions (see Appendix 1). Similar to the Finnish research design, we implemented a baseline assessment before the summer, a follow-up in the fall of the new school year, and a postassessment at the end of this school year. We extended the design by conducting the RCT for two school years, thus, with two additional assessments in the second school year. This allowed us to examine whether continued implementation of KiVa had effects after the first school year in which the teachers were trained. Antibullying lessons need to be implemented each year, specifically in Dutch primary schools that often have multigrade classrooms (e.g., combinations of grades 3/4 and 5/6), and group processes need to be guided again by teachers when the classroom composition changes. We translated the program and made some modifications before implementation (Appendix 2).

The first modification concerned the introduction of an additional intervention condition, the so-called KiVa+ condition. Twice per year (in November and June) teachers received feedback about the social relationships in their class (see Appendix 3 for an example, and Kaufman et al. 2020), based on the peer-nomination data for each wave. At the start of the RCT, there were indications (Sainio et al. 2011) that teachers at Finnish KiVa schools were able to identify only one out of four victims and that female victims were recognized less often than male victims (Haataja et al. 2016). The rationale behind providing schools feedback about the social structure of the class is that teachers will be better able to intervene. The feedback was provided through a report on the number of nominations children received for positive (friendships, like, popularity, leadership) and negative network questions (dislike, initiation of bullying as well as specific forms of bullying) and of their school wellbeing. To examine the effectiveness of KiVa and KiVa+, a three-armed research trial was conducted: KiVa, KiVa+, and the control group.

A second modification concerned indicated actions. Rather than using the confronting and nonconfronting approaches as applied in Finland (Garandeau et al. 2014b), we introduced a support group approach which is nonconfrontational but slightly different from the original approaches, because this approach emphasized the importance of the group (Van der Ploeg et al. 2016; see Appendix 1.2 for more details).

A third modification concerned the targeted grades. The KiVa lessons for the highest grades of primary education (unit 2) are targeted at grades 4–6 (Dutch grades 6–8). Because primary schools in the Netherlands often have multigrade classrooms (combinations of grades 3/4 and 5/6), we targeted KiVa unit 2 in grades 3/4, while allowing schools the opportunity to implement the program in grades 5/6. In grade 3 (with children aged 8 to 9 years), bullying is highly prevalent, and the processes that underlie bullying, victimization, and defending are comparable with those in higher grades (Veenstra et al. 2013).

We investigated whether KiVa reduced victimization and bullying during a 2-year period. We hypothesized that KiVa would lead to reductions in self-reported victimization (primary outcome measure) and bullying (most important secondary outcome measure). In addition, we expected stronger reductions of victimization and bullying in KiVa+ schools (compared with KiVa schools), because the detailed feedback reports would facilitate recognition of victims and stimulate teacher interventions.

## Method

### Sampling and Design

**Fig. 1:**
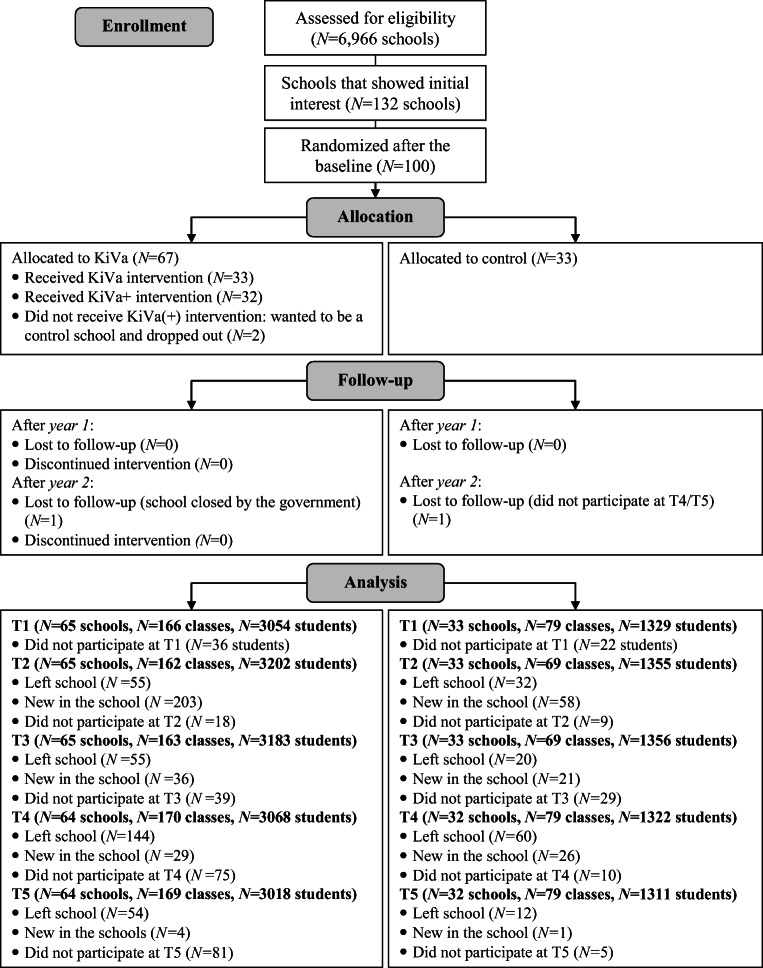
Flowchart of recruitment and retention of intervention and control schools

To recruit schools, we sent letters describing the KiVa project to all Dutch primary schools (*N* = 6966) in the fall of 2011, including information about the goals and content of KiVa, and an invitation to enroll in the RCT by filling in an online application form. A total of 132 schools indicated that they were willing to volunteer and were invited to participate in the baseline in May 2012. Some schools did not participate in the baseline because of lack of time or resources (e.g., computers) for the online questionnaires, or lack of commitment to implement an antibullying program. As a consequence, the number of 100 schools was slightly lower than what would be desired to reach 80% power (35 per condition, see Appendix 2.5). Children in grades 2–3 (ages 7–9, Dutch grades 4–5) from 100 schools completed the baseline (see Fig. 1).

After the baseline, the Netherlands Bureau for Economic Policy Analysis (CPB) randomly assigned the 100 schools to the control condition (33 schools) or to one of the two intervention conditions (34 in the KiVa condition and 33 in the KiVa+ condition; after the randomization, one KiVa and KiVa+ school dropped out), see Fig. 1. A stratified randomization procedure was used, based on a combination of the school averages of self-reported victimization, bullying, antibullying attitudes, and school well-being (see Appendix 2.6). Every stratum consisted of three schools with similar levels of bullying problems, from which each school was assigned to one condition. The top three schools with bullying problems and low school well-being were in the first stratum, the next three schools in the next stratum, and so on. As such, we tried to minimize differences between schools in the three conditions. KiVa was preregistered in the Netherlands Trial Register (https://www.trialregister.nl/trial/3903), but there was no preregistered trial protocol. See Appendix 4 for the CONSORT Checklist.

Principals and teachers who delivered KiVa-lessons received a 2-day training in June 2012 and started with the intervention at the beginning of the new school year in August 2012. KiVa schools were instructed to provide KiVa to 8- to 10-year-old children (grades 3 and 4; Dutch grades 5 and 6). Control schools were asked to continue their “care as usual” antibullying approach until their participation in the KiVa program in June 2014, which could be a variety of approaches, such as using an antibullying protocol, (a series of) freely available lessons from the internet, or a broader social-emotional learning program.

### Data Collection and Participants

Data collection took place in five waves: May 2012, October 2012, May 2013, October 2013, and May 2014. Students completed online questionnaires during regular school hours. This process was administered by the teachers, who received detailed instructions concerning the procedure prior to the data collection, and could request additional support through phone or e-mail. The order of questions and scales was randomized so that the order of presentation of the questions would not have any systematic effect on the results.

Students watched five short videos, all in a school setting, in which a professional actress explained the goal of the questionnaire (“investigating children’s well-being at school”), the way to fill out the questionnaire (including a sample question), and some basic rules, including that students were not allowed to talk to each other and to discuss their answers afterwards in order to ensure each other’s privacy. The videos also explained that individual answers would remain confidential, but that teachers would receive general feedback to improve the classroom climate. In a video, bullying was defined as formulated in the Olweus’ (1996) Bully/Victim questionnaire. Several examples covering different forms of bullying were given, followed by an explanation emphasizing the intentional and repetitive nature and the power difference between bullies and victims (bullies are stronger than victims, either physically or socially).

The intervention sample had 3309 students (49.9% boys, *M* age T1 = 8.66, *SD =* 0.70) in 65 schools, and the control sample had 1415 students (47.3% boys, *M* age T1 = 8.67, *SD* = 0.67) in 33 schools. The ethnic composition in intervention and control schools, respectively, was comparable, 78.6%/81.7% Dutch, 3.5%/1.7% Moroccan, 2.4%/1.5% Turkish, 2.4%/2.6% Surinamese, and 1.0%/1.1% Dutch Antilleans. The remaining 12.6%/11.4% of children reported another Western (6.5%/5.9%) or non-Western (5.6%/5.5%) ethnicity. Non-response rates were low (T1 = 1.3%; T2 = 0.6%; T3 = 1.5%; T4 = 1.9%; T5 = 2.0%), largely because data were collected digitally and students who incidentally missed the scheduled day of data collection could participate on another day within a month. The codebook has details on participants across waves, including participants in nontarget grades (Veenstra et al. 2020).

### Measures

Self-reported victimization and bullying (T1–T5) were measured with the global items from Olweus’ (1996) Bully/Victim questionnaire (victimization: “how often have you been victimized at school during the past couple of months?”; bullying: “how often have you bullied others at school during the past couple of months?”). We also assessed ten specific items concerning physical, verbal, relational, material, and cyber victimization/bullying. Children answered all items on a five-point scale 0 = “not at all”, 1 = “once or twice” (occasionally), 2 = “two or three times a month” (monthly), 3 = “about once a week” (weekly), and 4 = several times a week” (daily). The 10-item scale is commonly investigated by taking the mean score. The average implies that students who are bullied daily in one form, but are not being bullied in any other form, would receive a low score on this scale (i.e., 0.4). In other words, average scores mainly reflect the diversity of ways in which students are bullied, rather than the extent to which they are bullied. Because we aimed to investigate general reductions in bullying and victimization (rather than changes in specific forms), we used the maximum score given on any of the specific items (named hereafter victimization/ bullying maximum). Thus, a score of 4 would indicate that a student is bullied several times a week for at least one form of bullying. In additional logistic regression analyses, we dichotomized these responses to children who (were) bullied not at all or occasionally (0) and children who (were) bullied monthly, weekly, or daily (1) (see Solberg and Olweus 2003). Each of these 8 outcomes (i.e., the ordinal form and the logistic form of global bullying, global victimization, maximum bullying, and maximum victimization) was analyzed separately.

The dichotomous intervention variable indicated whether children were in the control (0) or KiVa condition (1). In additional analyses, we examined differences between KiVa (0) and KiVa+ (1). Boys and grade 4 were coded as 1 (girls and grade 3 as reference category).

### Analytical Strategy

We used longitudinal, cross classified, ordered multinomial logistic regressions in R2MLwiN to examine the effect of KiVa on self-reported victimization and bullying (Zhang et al. 2016), with Markov Chain Monte Carlo estimation (Browne 2017). Ordered logistic regressions can be applied to ordinal dependent variables, which fit the responses categories of the Olweus’ questionnaire (1996). In our models, the “non-victimized” group was the reference category.

Data collection was done in three consecutive school years; T1 in school year 2011–2012, T2 and T3 in school year 2012–2013, and T4 and T5 in school year 2013–2014. As Dutch primary schools often have multigrade classrooms, children can be in classrooms with a different student composition each school year. To account for this varying nesting structure, we applied cross classified models in which measurement waves and children were classified into (different) classrooms and schools (Browne 2017, chapter 15). Measurement wave was at the lowest level, and nested within children, classrooms, and schools. We assumed classroom stability within the school year and classified waves and children into the classroom structure of T1, T3, and T5 (we ignored that four children moved classrooms between T2 and T3, and 16 children moved classrooms between T4 and T5).

We used the effective sample size (ESS) as an indicator for convergence (Browne 2017, chapter 3). The ESS reflects the length of the Markov chain that is required to estimate a parameter or variance component. We considered the models as converged when the ESS was larger than 200 for all model parameters (Browne 2017). All models were estimated with a burn-in and iteration-rate of 400.000, and used orthogonal parameterization (Browne et al. 2009) with a thinning factor of 1. Scripts for all of our models can be found at https://osf.io/62zxf.

We included main effects for measurement wave (with T1 as reference category), intervention, and their interaction (wave *×* intervention). The effects of the intervention at a certain measurement wave can be obtained by adding the chain of estimates for the reference category (KiVa) to the chain of estimates for the interaction with time (KiVa *×* Tx). We included gender (individual level) and grade (individual level, because children can be in mixed-grade classrooms) as additional dichotomous variables, as well as their interaction with intervention to examine possible differences in intervention effects for gender or grade.

To examine the effects of the intervention on bullying and victimization, we examined intervention effects on the ordered global and maximum victimization and bullying outcomes using ordered multinomial logistic regressions (Tables 3 and 4). We also performed logistic regressions to examine whether the intervention reduced the number of children that report being a bully or victim (irrespective of the level of bullying or victimization, see Appendix 7), and we examined possible differences between KiVa and KiVa+ schools (see Appendix 8).

## Results

### Descriptive Statistics

We first examined the percentages of bullying and victimization at the five time points separately for control and intervention schools (see Table 1 for global victimization and Table 2 for global bullying). At the baseline, the total number of victims (referring to children who were victimized monthly, weekly, or daily) was comparable for control (33.4%) and intervention (35.0%) schools (Table 1). From T2 onwards, there was a clear decrease in the number of victimized students in both control and intervention schools, but the decrease was stronger in intervention schools. At T3, after one school year of implementation, the total number of victims reduced by 43.4% in intervention schools (compared with 26.2% at control schools). At T5, the reduction was 64.3% in intervention schools and 52.3% in control schools (a potential explanation for the large reduction at control schools is given in the “Discussion” section). Children’s maximum response to any of the ten combined forms of victimization (in Appendix 5, Table A5.1) followed a comparable pattern, although the initial prevalence for this measure of victimization was higher in both control and intervention schools.

**Table 1:** Self-reported global victimization in control and intervention schools in the focus cohort (grades 3–4)

	T1		T2		T3		T4		T5	
	N	%	N	%	N	%	N	%	N	%
Intervention	2994		3181		3133		2992		2935	
Not at all	1141	38.1	1463	46.0	1724	55.0	1945	65.0	1993	67.9
% change^1^						**+ 44.4**				**+ 78.2**
Once/twice	803	26.8	866	27.2	786	25.1	647	21.6	574	19.6
% change^1^						**− 6.3**				**− 26.9**
Monthly	291	9.7	257	8.1	227	7.2	141	4.7	147	5.0
% change^1^						**− 25.8**				**− 48.5**
Weekly	232	7.7	196	6.2	148	4.7	113	3.8	86	2.9
% change^1^						**− 39.0**				**− 62.3**
Daily	527	17.6	399	12.5	248	7.9	146	4.9	135	4.6
% change^1^						**− 55.1**				**− 73.9**
Total victims^2^	1050	35.0	852	26.8	623	19.8	400	13.4	368	12.5
% change^1^						**− 43.4**				**− 64.3**
Control	1287		1344		1326		1307		1306	
Not at all	512	39.8	620	46.1	652	49.2	758	58.0	793	60.7
% change^1^						**+ 23.6**				**+ 52.6**
Once/twice	345	26.8	351	26.1	347	26.2	318	24.3	305	23.4
% change^1^						**− 2.4**				**− 12.9**
Monthly	126	9.8	101	7.5	114	8.6	93	7.1	93	7.1
% change^1^						**− 12.2**				**− 27.3**
Weekly	73	5.7	88	6.5	86	6.5	51	3.9	41	3.1
% change^1^						**+14.3**				**− 44.7**
Daily	231	17.9	184	13.7	127	9.6	87	6.7	74	5.7
% change^1^						**− 46.6**				**− 68.4**
Total victims^2^	430	33.4	373	27.8	327	24.7	231	17.7	208	15.9
% change^1^						**− 26.2**				**− 52.3**

^1^The percentages of change are calculated relative to the T1 prevalence

^2^The sum of victims who are victimized monthly, daily, or weekly. Entries highlighted in bold refer to the most important numbers in this table

**Table 2:** Self-reported global bullying in control and intervention schools in the focus cohort (grades 3–4)

	T1		T2		T3		T4		T5	
	N	%	N	%	N	%	N	%	N	%
Intervention	2993		3180		3131		2992		2933	
Not at all	1934	64.6	2291	72.0	2404	76.8	2422	80.9	2453	83.6
% change^1^						**+ 18.8**				**+ 29.4**
Once/twice	727	24.3	638	20.1	531	17.0	444	14.8	397	13.5
% change^1^						**− 30.2**				**− 44.3**
Monthly	148	4.9	109	3.4	101	3.2	66	2.2	42	1.4
% change^1^						**− 34.8**				**− 71.0**
Weekly	71	2.4	58	1.8	34	1.1	24	0.8	22	0.8
% change^1^						**− 54.2**				**− 68.4**
Daily	113	3.8	84	2.6	61	1.9	36	1.2	19	0.6
% change^1^						**− 48.4**				**− 82.8**
Total bullies^2^	332	11.1	251	7.9	196	6.3	126	4.2	83	2.8
% change^1^						**− 43.6**				**− 74.5**
Control	1279		1344		1325		1304		1306	
Not at all	858	67.1	983	73.1	954	72.0	1012	77.6	1006	77.0
% change^1^						**+ 7.3**				**+ 14.8**
Once/twice	294	23.0	250	18.6	283	21.4	238	18.3	259	19.8
% change^1^						**− 7.1**				**− 13.7**
Monthly	48	3.8	48	3.6	44	3.3	24	1.8	22	1.7
% change^1^						**− 11.5**				**− 55.1**
Weekly	28	2.2	23	1.7	18	1.4	16	1.2	12	0.9
% change^1^						**− 37.9**				**− 58.0**
Daily	51	4.0	40	3.0	26	2.0	14	1.1	7	0.5
% change^1^						**− 50.8**				**− 86.6**
Total bullies^2^	127	9.9	111	8.3	88	6.6	54	4.1	41	3.1
% change^1^						**− 33.1**				**− 68.4**

^1^The percentages of change are calculated relative to the T1 prevalence

^2^The sum of bullies who bully monthly, daily, or weekly. Entries highlighted in bold refer to the most important numbers in this table

For bullying, the total number of children who reported bullying others at least monthly was 11.1% in intervention schools and 9.9% in control schools at the baseline (Table 2). From T2 onwards, there appeared a decreasing trend in the number of self-reported bullies in both control and intervention schools. At T3, self-reported bullying decreased by 43.6% in intervention schools, compared with the 33.1% reduction in control schools. At T5, the reduction was 74.5% in intervention schools and 68.4% in control schools. There were clearly more children who indicated that they bullied others with the maximum response to any of the ten combined forms of bullying than with the global item (Appendix 5, Table A.5.2).

### Intervention Effects with Ordered Multinomial Regressions

The ordered logistic regressions tested the effects of KiVa while accounting for the nested data structure (see Table 3). The negative intercepts indicate that over all five waves, reporting occasionally, monthly, weekly, or daily victimization or bullying was less likely than reporting being nonvictimized (the reference category). Over time, the prevalence of victimization decreased significantly in control schools (see effects for T2, T3, T4, and T5). There were no significant differences between KiVa and control schools at the baseline (effect: KiVa) or at T2 (effect: KiVa × T2), but from T3 onwards, KiVa-schools had significantly stronger reductions in self-reported victimization than control schools (interactions of KiVa with T3, T4, and T5 for both the global and maximum victimization measures). The interpretation of the intervention effects on victimization at T3 and T5 (after one and two school years of implementation) were obtained by summing the KiVa-effect and its interaction with time (see Table 4). At T3, the difference between control schools and intervention schools in frequency of global and maximum victimization did not reach significance, although effects were in the expected direction (OR_Global_ = 0.77, *p* = 0.118; 95% CI [0.56; 1.07]; OR_Maximum_ = 0.74, *p* = 0.061, 95% CI [0.54; 1.01]). At T5, the difference between intervention and control schools was significant for the maximum score, but not the global score (OR_Global_ = 0.74, *p* = 0.081, 95% CI [0.54; 1.04]; OR_Maximum_ = 0.66, *p* = 0.01, 95% CI [0.48; 0.91]). The inverse odds ratios (1/OR) can help to interpret the strength of the effects (see Table 4): for global victimization, the odds for KiVa students to be victimized were 1.29 and 1.34 lower than for control students, 1 year and two school years after the intervention started (for the maximum score 1.35 and 1.52).

**Table 3:** Multinomial logistic regressions to estimate the effects of the intervention on self-reported victimization and bullying

	Victimization (global)				Victimization (maximum)				Bullying(global)				Bullying (maximum)			
	Est.	SE	OR	*p*	Est.	SE	OR	*p*	Est.	SE	OR	*p*	Est.	SE	OR	*p*
Intercept																
2: Occasionally	0.85	(0.14)	2.34	< 0.001	1.71	(0.14)	5.50	< 0.001	− 1.28	(0.16)	0.28	< 0.001	− 0.65	(0.16)	0.52	< 0.001
3: Monthly	− 0.87	(0.14)	0.42	< 0.001	0.04	(0.13)	1.04	0.76	− 3.40	(0.17)	0.03	< 0.001	− 2.70	(0.17)	0.07	< 0.001
4: Weekly	− 1.57	(0.14)	0.21	< 0.001	− 0.91	(0.13)	0.40	< 0.001	− 4.17	(0.17)	0.02	< 0.001	− 3.46	(0.17)	0.03	< 0.001
5: Daily	− 2.22	(0.14)	0.11	< 0.001	− 1.60	(0.14)	0.20	< 0.001	− 4.77	(0.17)	0.01	< 0.001	− 3.96	(0.17)	0.02	< 0.001
KiVa	0.19	(0.16)	1.21	0.238	0.22	(0.16)	1.24	0.172	0.11	(0.19)	1.11	.587	0.06	(0.20)	1.06	.774
Boy	− 0.08	(0.11)	0.92	0.438	− 0.09	(0.11)	0.92	0.414	0.91	(0.11)	2.48	< 0.001	0.83	(0.11)	2.29	< 0.001
Grade 4	− 0.20	(0.14)	0.82	0.148	− 0.33	(0.13)	0.72	0.012	− 0.08	(0.14)	0.92	0.559	− 0.30	(0.14)	0.74	0.035
KiVa × Grade 4	− 0.21	(0.16)	0.81	0.197	− 0.11	(0.16)	0.90	0.492	0.10	(0.17)	1.10	0.571	0.23	(0.17)	1.26	0.175
KiVa × Boy	0.01	(0.13)	1.01	0.953	0.02	(0.13)	1.02	0.854	− 0.17	(0.13)	0.85	0.202	− 0.16	(0.13)	0.85	0.216
Change wave 2																
T2	− 0.38	(0.08)	0.68	< 0.001	− 0.43	(0.08)	0.65	< 0.001	− 0.40	(0.10)	0.67	< 0.001	− 0.56	(0.09)	0.57	< 0.001
KiVa × T2	− 0.14	(0.10)	0.87	0.152	− 0.17	(0.09)	0.85	0.071	− 0.10	(0.12)	0.91	0.399	− 0.01	(0.11)	0.99	0.893
Change wave 3																
T3	− 0.63	(0.08)	0.53	< 0.001	− 0.78	(0.08)	0.46	< 0.001	− 0.39	(0.10)	0.68	< 0.001	− 0.76	(0.09)	0.47	< 0.001
KiVa × T3	− 0.45	(0.10)	0.64	< 0.001	− 0.52	(0.09)	0.59	< 0.001	− 0.46	(0.12)	0.63	< 0.001	− 0.46	(0.11)	0.63	< 0.001
Change wave 4																
T4	− 1.17	(0.08)	0.31	< 0.001	− 1.40	(0.08)	0.25	< 0.001	− 0.82	(0.10)	0.44	< 0.001	− 1.06	(0.10)	0.34	< 0.001
KiVa × T4	− 0.50	(0.10)	0.61	< 0.001	− 0.50	(0.10)	0.61	< 0.001	− 0.38	(0.12)	0.68	0.002	− 0.48	(0.12)	0.62	< 0.001
Change wave 5																
T5	− 1.35	(0.09)	0.26	< 0.001	− 1.58	(0.08)	0.21	< 0.001	− 0.84	(0.10)	0.43	< 0.001	− 1.27	(0.10)	0.28	< 0.001
KiVa × T5	− 0.49	(0.10)	0.61	< 0.001	− 0.64	(0.10)	0.53	< 0.001	− 0.62	(0.12)	0.54	< 0.001	− 0.56	(0.12)	0.57	< 0.001
Variance																
School level	0.08	(0.03)			0.09	(0.04)			0.24	(0.06)			0.27	(0.07)		
Classroom level T1	0.11	(0.05)			0.06	(0.04)			0.13	(0.06)			0.13	(0.06)		
Classroom level T3	0.05	(0.05)			0.07	(0.05)			0.06	(0.05)			0.06	(0.05)		
Classroom level T5	0.03	(0.03)			0.04	(0.04)			0.02	(0.03)			0.02	(0.03)		
Student level	2.69	(0.10)			2.82	(0.10)			2.15	(0.11)			2.46	(0.12)		

*Est.* estimate

**Table 4:** Overview of odds ratio’s and confidence intervals for the intervention effects compared with the control schools

	Frequency (multinomial logistic regressions)				Occurrence (binomial logistic regressions)			
	OR	*p*	95% CI	Inverse OR	OR	*p*	95% CI	Inverse *OR*
Victimization								
Global, T3^1^	0.77	0.118	0.56–1.07	1.29	0.67	0.031	0.46–0.97	1.49
Global, T5^2^	0.74	0.081	0.54–1.04	1.34	0.71	0.086	0.49–1.05	1.40
Maximum, T3	0.74	0.061	0.54–1.01	1.35	0.70	0.052	0.49–1.00	1.43
Maximum, T5	0.66	0.011	0.48–0.91	1.52	0.61	0.010	0.42–0.89	1.63
Bullying								
Global, T3	0.70	0.069	0.48–1.03	1.43	0.84	0.501	0.51–1.38	1.19
Global, T5	0.60	0.011	0.40–0.89	1.67	0.83	0.538	0.47–1.49	1.20
Maximum, T3	0.67	0.047	0.45–1.00	1.49	0.70	0.165	0.42–1.16	1.41
Maximum, T5	0.60	0.015	0.40–0.91	1.66	0.65	0.009	0.35–1.08	1.63

^1^T3 is one school year after implementing the intervention

^2^T5 is two school years after implementing the intervention

Table 3 also provides results for self-reported global bullying. The majority of children did not report bullying others, as indicated by the negative intercepts. Self-reported bullying decreased over time (effects for T2, T3, T4, and T5), and there were additional KiVa-effects on the reduction of bullying (interactions of KiVa with T3, T4, and T5). The combination of effects in Table 4 shows that at T3, the intervention effect was not or just significant (OR_Global_ = 0.70, *p* = .069, 95% CI [0.48; 1.03]; OR_Maximum_ = 0.67, *p* = 0.047, 95% CI [0.45; 1.00]), but at T5, bullying was significantly lower in intervention schools than in control schools (OR_Global_ = 0.60, *p* = 0.011, 95% CI [0.40; 0.89]; OR_Maximum_ = 0.60, *p* = 0.015, 95% CI [0.40; 0.91]). The inverse odds ratios indicated that the odds of reporting being a bully for KiVa students were 1.67 lower than the odds for control students at T5 (1.66 for the maximum score).

### Gender and Grade

Table 3 shows that there was no significant difference in the level of self-reported victimization between boys and girls (effect: boy), but maximum victimization was significantly lower in grade 4 compared with grade 3. There were also no significant interaction-effects of KiVa with either gender or grade on victimization. Boys indicated more often than girls that they bullied. There were no significant differences between global bullying in grades 3 and 4, but maximum bullying was significantly lower in grade 4 compared to grade 3. Table 3 further showed no significant interaction-effects of KiVa with either gender or grade on bullying. Given our large sample, we therefore assume that differences in KiVa-effects between boys and girls or children in grade 3 or 4 are absent or very small.

### Logistic Regressions

To examine the effectiveness of the intervention, not only the frequency of bullying or victimization but also the number of students who indicate that they bully or are victimized regularly is important. We used binomial logistic regressions to compare students who indicate victimization/bullying monthly or more often with the other students. The results are in Appendix 7, Table A7.1 and in Table 4, and indicate significant and consistent effects of the intervention on self-reported levels of victimization (all inverse ORs between 1.40 and 1.63). The relative risk ratio for victimization at T5 in intervention schools as compared to control schools was 75% for the global and 71% for the maximum score. The effects for bullying were in the expected direction, but only reached significance for the maximum bullying score at T5 (inverse ORs between 1.19 and 1.63). The relative risk ratio at T5 for the maximum bullying score at intervention schools was 69%.

### KiVa+

Additional analyses with multinomial models (Tables in Appendix 6) and logistic models (Tables A7.2 and A7.3) showed no significant differences between KiVa and KiVa+, either for victimization or for bullying, with wide confidence intervals for the OR containing 1 in each model (see also Appendix 8). These findings provide no reason to assume that the effectiveness differs between KiVa and KiVa+.

## Discussion

KiVa is a systematic, school-wide antibullying program that targets students, group processes, teachers, and the school as a whole. KiVa initially showed evidence of effectiveness in reducing bullying and victimization in Finland (Kärnä et al. 2011b). It is important to demonstrate whether KiVa is effective outside Finland. With this aim, we evaluated the KiVa antibullying program in the Netherlands in a randomized controlled trial (RCT) involving 98 schools with 4383 students. Victimization and bullying decreased at Dutch KiVa schools, and this decrease was significantly stronger than in control schools. After one school year of implementing the intervention, all effects were in the expected direction, but mostly nonsignificant, but after two school years, clearly and mostly significant intervention effects were found. Overall, there was no evidence for adverse effects that indicate that intervention schools did worse than control schools. Thus, KiVa’s effectiveness has been replicated first in Italy (Nocentini and Menesini 2016) and now in the Netherlands.

The intervention effects were more pronounced for the frequency of bullying (children’s complete response patterns with multinomial logistic regressions), than for the number of students involved in bullying (dichotomized answers with logistic regressions). For victimization, this pattern was reversed. It is important to realize that conclusions about the effectiveness of an intervention may differ depending on whether either the frequency or the number of children involved is examined. We argue that examining the full range of responses using multinomial regressions is better than analyzing dichotomized answers, because this allows researchers to detect, as is the case in our study, whether some children may still bully after the intervention, while the frequency of their bullying may be decreased.

### Modifications in the Dutch Implementation

The data in the present RCT did not indicate that attempts to improve signaling of victims with an additional component (KiVa+) resulted in a higher effectiveness. Of course, replication is needed, and false negative results are possible. Yet, given our large sample, we have currently no reason to assume that providing teachers with reports on the social position of students has additional effects on reducing bullying. This is surprising, because teachers in the regular KiVa condition often requested further information about students. Our findings suggest that it requires more than merely providing teachers with feedback to further reduce bullying and victimization. In a new study, we will ask teachers to translate the feedback into a specific action plan, in particular for persistent victims (see Kaufman et al. 2018, 2020).

Another modification was that we only offered schools the nonconfronting support group approach as indicated action. Although not analyzed in the current study, a small-scale evaluation on 38 victims for whom a support group was organized did not show that this approach reduced long-term victimization (Van der Ploeg et al. 2016). Recent Finnish research on the experiences of 341 bullies participating in indicated actions suggest that a combination of confronting (e.g., blaming bullies specifically for their behavior) and nonconfronting elements (e.g., increasing empathy for victims) might be optimal (Garandeau et al. 2018b). Bullies’ intention to change behavior was highest when empathy arousal and condemning of the behavior were high. The combination might be included and tested in future approaches.

We introduced materials that were originally developed for grades 4–6 in grades 3 and 4. The findings showed no evidence for differential effectiveness between grades; none of the interaction terms between KiVa and grade reached significance, and there was no clear pattern of results across analyses. Thus, an earlier start of the program in the Netherlands than in Finland did not seem to affect the effectiveness of the program, but instead helped to reduce bullying already one school grade earlier. We also found no significant gender differences in the effectiveness of the intervention. As an earlier study also found no moderation of KiVa effects by gender and age (Kärnä et al. 2011b), our results may indicate that the effectiveness of KiVa in upper grades of primary schools does not depend on gender or grade.

### Unique Societal Circumstances During the RCT

Shortly after the start of the evaluation of KiVa in the Netherlands, bullying became a topic of national concern due to the suicides of three teenagers, Tim Ribberink (November 2012), Fleur Bloemen (December 2012), and Annas Aouragh (February 2013), allegedly because they were victims of bullying. These suicides led to a lot of media attention for (prevention of) bullying and to public pressure to take action. The Ministry of Education initiated new regulations for schools, and many youth television programs and magazines paid attention to the mechanisms behind bullying. As a result, these incidents may have influenced the experimental setup and motivated both intervention and control schools to take action.

Control schools also made reductions in bullying and victimization, with 26% fewer victims after the first school year and 52% after the second school year in the RCT. Absolute differences in victimization between KiVa and control schools were smaller in the Netherlands (17% after year 1, 12% after year 2) than in Finland (29%). However, KiVa schools may have benefited from the national debate too, resulting in a larger absolute reduction of victimization at KiVa schools in the Netherlands (decrease of 43% after 1 year of implementation—and 64% after 2 years) than in Finland (40% decrease after 1 year). Despite the national debate about victimization and alleged additional efforts of control schools, larger reductions in bullying and victimization were found in KiVa schools compared with control schools. The effect sizes are small but comparable with those of other antibullying programs (Gaffney et al. 2018).

### Limitations, Strengths, and Future Directions

The Dutch implementation of KiVa in 2012 was the first dissemination of KiVa in another country. Implementation and evaluation designs were highly comparable with those in Finland (although extended with a second evaluation year), and the core components of the KiVa program, the measurement instruments as well as the timing of assessments were kept the same. Strengths of our study are the independent evaluation from the program developers, the longitudinal time frame and long-term evaluation (five waves in 2 years), and the large sample size with high retention rates with more than 250 target classrooms from 98 schools.

The sample was not representative for the Dutch population. Although all Dutch schools were invited to participate in the research, all intervention and control schools volunteered to do so. Schools from all of the 12 Dutch provinces were represented, from rural to suburban and urban areas. There were, however, relatively more schools participating from the Northern provinces (48%), probably because the University of Groningen is located in the North of the Netherlands. About 46% of the schools had a religious denomination, which is lower than the Dutch average of 62% (versus 38% that offers public education). The mean number of students per school was 215, which is close to the mean in Dutch elementary schools of 218. The findings are, thus, generalizable to schools in the Netherlands that are motivated and willing to implement a school-wide antibullying program.

We investigated intervention effects on self-reported bullying and victimization. Although self-reports capture specific experiences often not observed by others, they are also sensitive to potential biases, because children may underreport bullying, overreport victimization, or may not be willing to report painful experiences. Peer reports, however, also come with their limitations, because these may be sensitive to prejudices and reputations (Olweus 2010), and reputations may remain even after bullying has stopped.

A limitation is that we did not account for the implementation fidelity in our analyses. Information on implementation was limited, because not all schools returned the requested implementation portfolios at the end of the second implementation year. The Finnish evaluation showed variation in implementation profiles of teachers (Haataja et al. 2015), and adherence to the lessons was a positive predictor for decreases in victimization (Haataja et al. 2014; Swift et al. 2017).

Finally, despite the success of the intervention, there are indications of persistent cases. KiVa was less effective for popular bullies in Finland (Garandeau et al. 2014a). In addition, 3.6% of Dutch children at KiVa schools were persistent victims, who remained victimized for 2 years (Kaufman et al. 2018). This calls for further research and intervention development, because victimization consequences may be more severe for victims in schools with highly salient antibullying efforts (Garandeau et al. 2018a; Huitsing et al. 2019), because these victims have fewer others with whom to share their plight, and they tend to engage more in self-blaming attributions.

Taken together, our findings indicate that the KiVa antibullying program is successful in decreasing victimization and bullying in the Netherlands, with small effect sizes that are comparable with KiVa effects in other countries and the effects of other interventions.

## Electronic supplementary material

ESM 1 (DOCX 110 kb)
