# Supplementary material for: A Large-Scale Replication of the Effectiveness of the KiVa Antibullying Program: a Randomized Controlled Trial in the Netherlands
Source: Prev Sci. 2020 May 12;21(5):627–38. doi: 10.1007/s11121-020-01116-4 (PMC7305071; doi:10.1007/s11121-020-01116-4)
Supplement: Supplementary file 1 — (DOCX 110 kb) [file 11121_2020_1116_MOESM1_ESM.docx]

**Appendices to:**

**A Large-Scale Replication of the Effectiveness of the KiVa Antibullying Program: A Randomized Controlled Trial in the Netherlands**

[Appendix 1: KiVa Components 2](#_Toc23761484)

[Appendix 2: Implementing KiVa in the Netherlands 7](#_Toc23761485)

[Appendix 3: Example of KiVa+ feedback to teachers 10](#_Toc23761486)

[Appendix 4: CONSORT 2010 Checklist 12](#_Toc23761487)

[Appendix 5: Absolute and Relative Numbers of Victims and Bullies (Maximum Scores) 15](#_Toc23761488)

[Appendix 6: Multinomial Logistic Regressions for KiVa and KiVa+ 17](#_Toc23761489)

[Appendix 7: Logistic Regressions 19](#_Toc23761490)

[Appendix 8: Overview of ORs and CIs for the KiVa and KiVa+ Effects 22](#_Toc23761491)

[References 23](#_Toc23761492)

# Appendix 1: KiVa Components


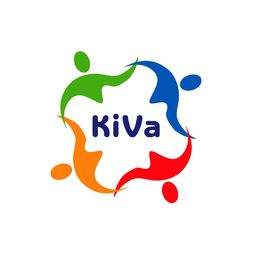


The KiVa intervention shares some features with other school-wide approaches, such as the Olweus program and Steps to Respect, but KiVa also presents some unique components. KiVa includes a broad array of concrete and professionally prepared materials for teachers and students. Moreover, while focusing on bystanders, KiVa goes beyond emphasizing their role; it provides ways to enhance students’ and teachers’ attitudes, empathy, self-efficacy, and efforts to support victims (Saarento, Boulton, & Salmivalli, 2015). The general goal of KiVa is to create schools where bullying is unacceptable, and all students and teachers are encouraged to contribute to this goal. Below, the components of the intervention are listed.

**1. Universal Components**

**a. Two days of face-to-face training for school personnel**. During the KiVa START-training, teachers learn about bullying and related group dynamics. They are also instructed to be aware of what happens during school breaks, physical education lessons, or after school. The first training day is about giving the KiVa lessons and conducting group discussions. The second day is about solving bullying with indicated actions if it happens, despite the prevention.

**b. Manualized theme-lessons.** There are concrete theme-lessons for students, delivered by teachers. The topics of the theme-lessons proceed from general topics, such as the importance of respect in relationships, group communication, and group pressure, to bullying and its mechanisms and consequences. The aims of the lessons are to raise awareness of the role that the group plays in maintaining bullying, to increase empathy with victims, to promote children’s strategies for supporting victims and thus their self-efficacy to do so, and to increase children’s coping skills when victimized. Teachers are asked to give one 90-minute KiVa lesson per month. The ten theme-lessons involve discussions, group and role-play exercises, and short films about bullying. For the implementation in Dutch schools, some modifications were made in some pictures and stories to fit the Dutch context. For the second implementation year, teachers received some additional exercises that they can implement when teaching multigrade classrooms (implying that about half of the classroom participated for the second time in the KiVa lessons).

**c. Virtual learning environment for students.** Students practice their antibullying skills during and between the lessons in a virtual learning environment designed as an interactive computer game. The game involves five levels and the teacher activates a level as soon as particular lessons have been given. In each level, the children: (a) can learn new facts about bullying and examine what they have learned from the lessons thus far; (b) can move around in a virtual school and face different challenges and decide how to respond, what to say and to do. They receive feedback based on their choices and can examine the feelings and thoughts of the characters in the game; (c) are asked to report what they have put into practice. For instance, whether they have treated others with respect, resisted group pressure, or supported a victim. Again, the children receive personalized feedback based on their reports.

**d. Parental involvement**. Parents receive a leaflet that includes information about the KiVa program and a link to an online guide with information about bullying and advices about how to recognize it and what to do if their child is a victim or a bully. School staff receives an extensive PowerPoint presentation as material for a parent evening at the school.

**e. KiVa symbols**. Other universal components include vests for the recess supervisors and posters to remind students and school personnel about the KiVa program.

**2. Indicated actions**

In KiVa-schools a team of at least three staff members receive training to address each case of bullying witnessed or revealed. In the Dutch schools, cases of bullying are addressed through a specific procedure involving a series of discussions: first, an individual meeting with the victim, then a group discussion with the bully (or bullies) and some pro-social classmates to enhance support toward the victim. These discussions take place immediately after the case of bullying has come to the attention of the team. About 1 or 2 weeks afterward, follow-up meetings are organized with the victim and the support group separately in order to monitor the possible expected changes (see: Van der Ploeg, Steglich, & Veenstra, 2016).

**a. The support group approach.** In the Netherlands, the Support Group Approach was implemented, and this approach differs from the confronting/non-confronting approach in Finland (see: Garandeau, Poskiparta, & Salmivalli, 2014). In line with the theoretical insights behind KiVa, the Support Group Approach is based on the idea that bullying is a group phenomenon and that others can alter bullies’ motivations to bully (Rigby, 2014; Robinson & Maines, 2008; Young, 1998). Instead of focusing only on (changing) the behavior of the bully, bystanders and defenders are also involved in tackling bullying situations. The purpose of the support group is not to punish or blame bullies and their assistants, but to create mutual concern for the well-being of the victim. It is emphasized that everyone has to do something to help to improve the situation. In other words, the responsibility to solve bullying is given to the support group. It is assumed that the shared distress will evoke empathy within the bullies and that the ‘group pressure’ of shared responsibility will trigger bullies’ willingness to alter their behavior. Moreover, assistants are expected to lose the excitement and arousal of watching bullying.

**3: TIDieR Checklist (Hoffman et al., 2014)**

| **Brief name** | **Item** | **Where located  (Paper or Appendix)** |
| --- | --- | --- |
| **1** | Provide the name or a phrase that describes the intervention Describe | 🗹 First paragraph of *KiVa antibullying program* |
| **Why** |  |  |
| **2** | Provide the name or a phrase that describes the intervention Describe any rationale, theory, or goal of the elements essential to the intervention | 🗹 Second and third paragraph of *KiVa antibullying program* (e.g., “Three insights provide support for the rationale of the group […]” |
| **What** |  |  |
| **3** | Materials: Describe any physical or informational materials used in the intervention, including those provided to participants or used in intervention delivery or in training of intervention providers. Provide information on where the materials can be accessed (such as online appendix, URL) | 🗹 See *Appendices 1 and 3* Appendix 1 (all KiVa components) is referred to in the *Kiva Antibullying Program* paragraph, and Appendix 3 (example of feedback report for teachers) is referred to in the *The Present Study* section. |
| **4** | Procedures: Describe each of the procedures, activities, and/or processes used in the intervention, including any enabling or support activities | 🗹 See *Sampling and Design*, which also refers to *Appendix 2* (Implementing KiVa in the Netherlands). |
| **Who provided** |  |  |
| **5** | For each category of intervention provider (such as psychologist, nursing assistant), describe their expertise, background, and any specific training given | 🗹 See *Appendix 2.7*, referred to in the *Sampling and Design* section, and in the introduction section (description of the START training) |
| **How** |  |  |
| **6** | Describe the modes of delivery (such as face to face or by some other mechanism, such as internet or telephone) of the intervention and whether it was provided individually or in a group | 🗹 See *Appendix 1*, referred to in the *KiVa antibullying program* section. |
| **Where** |  |  |
| **7** | Describe the type(s) of location(s) where the intervention occurred, including any necessary infrastructure or relevant features | 🗹 See *2^nd^ paragraph* (“The KiVa anti-bulling program is such a school-wide approach”) |
| **When and How Much** |  |  |
| **8** | Describe the number of times the intervention was delivered and over what period of time including the number of sessions, their schedule, and their duration, intensity, or dose | 🗹 See *The Present Study* (“We implemented the core components of KiVa that […[”) and *Appendix 1* (referred to in the *Kiva antibullying program* section). |
| **Tailoring** |  |  |
| **9** | If the intervention was planned to be personalised, titrated or adapted, then describe what, why, when, and how If | Not applicable |
| **Modifications** |  |  |
| **10** | If the intervention was modified during the course of the study, describe the changes (what, why, when, and how)  Planned: | 🗹 See *The Present Study* (three aspects are important) |
| **How well** |  |  |
| **11** | Planned: If intervention adherence or fidelity was assessed, describe how and by whom, and if any strategies were used to maintain or improve fidelity, describe them  Actual: | 🗹 See *Discussion*, included as a limitation |
| **12** | Actual: If intervention adherence or fidelity was assessed, describe the extent to which the intervention was delivered as planned | Not applicable |

# Appendix 2: Implementing KiVa in the Netherlands

Several actions were taken to facilitate the implementation of KiVa in the Netherlands.

**1. KiVa consortium**. A KiVa consortium was established that comprises scholars and people who are working in the field of education (e.g., professional school trainers). Together they meet in monthly meetings (since January 2011, to now) to discuss the practical implementation of KiVa and the scientific evaluation. The collaboration of scientists and practitioners in the KiVa consortium contributes significantly to a solid implementation of the intervention.

**2. Translating materials**. All Finnish teaching materials were translated into Dutch. The translation process started in January 2011 and finished in October 2012. The time span of translating all the materials was rather long in order to further improve the KiVa materials and to adjust the Finnish material to the Dutch educational context.

**3. Online data collection**. An online tool was developed to facilitate the data collection. The development of the questionnaire started in September 2011. The questionnaire was largely based on the questionnaire from Finland, but also contained several added measurement scales to measure for instance social goals or parenting practices. The online questionnaire was finalized in April 2012 after several pilots, both at small scale (e.g., talking with children about their experiences) and large scale (e.g., a complete pilot school).

**4. Recruiting schools**. In October 2011, the website www.kivaschool.nl was launched and a letter was sent to all schools in the Netherlands to inform them about the opportunity of participating in the evaluation of the effectiveness of KiVa. The schools were invited to participate for free. Schools who wanted to participate could register through the website. In the registration form, some questions were asked about the anti-bullying actions of the schools. It appeared that one third of the schools already had an anti-bullying program, but they wanted something new. The registration period stopped in April 2012. The schools received information about the research design, the upcoming baseline assessment in May 2012, and KiVa START-training in June 2012.

**5. Power calculations**. With the program Optimal Design (Spybrook et al., 2011), the required number of schools has been calculated. The design chosen has students nested within classes within schools, with randomization at school level. In determining the optimum number of schools, we assumed a significance level (α) of 0.05, 25 students per class (s), 3 classes per school (Y), a variance between schools of 4% and between classes of 12%, an effect size (δ) of 0.20 and the inclusion of aggregated covariates at school level that explain 50% of the variance. These figures were taken from Finland (Kärnä et al., 2011).

To obtain a power of 80% at the student level, it was necessary to include 35 control schools and 35 intervention schools per condition. The total number of schools with a pairwise comparison is then 70, see the figure below. For our research with two intervention conditions, this means 105 schools with 315 classes. Because some schools that indicated initial interest dropped out, the final sample of 98 schools was slightly lower than the desired number of schools.


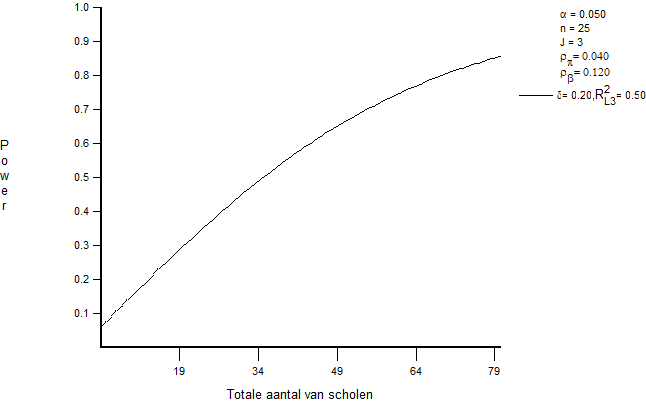


Total number of schools

**6. Randomization**. After the baseline assessment, a stratified randomization procedure was used, based on a combination of the following four measures. The first two measures were self-reported victimization and bullying. This was measured using the Olweus Bully/Victim questionnaire (1996). This scale consists of 1 global item and 10 specific items concerning several forms for both bullying and victimization, that measures the frequency of bullying on a 5-point Likert-scale, ranging from 0 “It did not happen” to 4 “Several times per week”. An example item for victimization is “I was hit, kicked or pushed”, an example item for bullying is “I hit, kicked or pushed someone”. The third measure was antibullying attitudes (Kärnä et al., 2011; Rigby & Slee, 1991). Participants responded to 8 items (e.g., “It is okay to call some kids nasty names”) on a 5-point scale ranging from 1 “never true” to 5 “always true”. The fourth scale was school well-being ( Kärnä et al., 2011). Participants responded to 7 items (e.g., “I like it at school”) on a 4-point Likert scale ranging from 1 “never” to 4 “always”. These items were reverse coded.

We computed the school averages of these measures and transformed them to standardized *z*-scores, after which we added the four standardized outcomes to one measure that reflect the level of “bullying-related problems” at schools. After constructing these scores, we send these scores to the Netherlands Bureau for Economic Policy Analysis (CPB), who executed the stratified randomization procedure and randomly assigned schools to the control condition or to one of the two intervention conditions.

**7. Teacher training program.** Teachers, principals, and school counselors were invited for the START-training in June 2012. The training took place in groups of about 25 participants. These groups were trained by duos, composed of a professional school trainer and a scientist from the Dutch research group. The training program was mainly developed by school trainers and repeatedly discussed during the monthly KiVa consortium meetings. In April and May 2012, train-the-trainer days familiarized professional trainers with KiVa.

# Appendix 3: Example of KiVa+ feedback to teachers

Table 3.1. *Example of Network Feedback to Teachers in the KiVa+ condition*

|  | Network nominations | | | | | | | Self-report | |
| --- | --- | --- | --- | --- | --- | --- | --- | --- | --- |
|  | Bullying | Social preference | | | Social reputation | | | Victimization | Well-being |
|  | 1. | 2. | 3. | 4. | 5. | 6. | | 7. | 8. |
| Student | Bully | Best friend | Like | Dislike | Popular | Leader | | Yes/no | Level |
| Class average | 0.36 | 2.65 | 6.31 | 2.69 | 3.42 | 2.73 | | -- | -- |
| ***Girls*** |  |  |  |  |  |  |  |  |  |
| Liz |  | ***0*** | ***0*** | ***8*** | 1 | 2 | |  |  |
| Anna | ***3*** | 5 | ***10*** | 2 | ***8*** | ***8*** | |  |  |
| Meg |  | 3 | 6 | 1 | ***6*** | 2 | |  |  |
| Cho |  | 3 | 7 | 2 | ***4*** | ***9*** | |  |  |
| Jazz |  | 5 | 8 | 2 | ***5*** | ***7*** | |  |  |
| Debby |  | 6 | 8 | 2 | ***6*** | 5 | |  |  |
| Roxanne |  | 3 | 7 | 2 | 1 | 2 | |  |  |
| Sarah |  | 2 | 7 | 1 | 2 | 1 | |  | **Low** |
| Sophie |  | ***0*** | ***2*** | 4 | 1 | 1 | | **Yes** | **Very Low** |
| Nyen |  | 1 | 6 |  |  | 1 | |  |  |
| Emma |  | 2 | ***11*** | 3 | 2 | 2 | |  |  |
| Olivia |  | 3 | ***10*** | 2 | 2 | 3 | |  |  |
| ***Boys*** |  |  |  |  | 2 |  | |  |  |
| Jacob |  | 3 | ***3*** | 2 | 1 | 1 | |  |  |
| Mason |  | 1 | 6 | 2 | 2 | 2 | |  | **Low** |
| William |  | 2 | ***10*** | 3 | 1 | 1 | |  |  |
| Jayden |  | 4 | ***10*** | 1 |  | ***9*** | |  |  |
| Isaac | ***2*** | 3 | ***3*** | ***9*** | ***7*** | 2 | |  |  |
| Ethan | ***2*** | 3 | ***3*** | ***11*** | ***8*** | 2 | |  | **Very Low** |
| Elijah | ***2*** | 4 | ***3*** | 2 | ***8*** | 1 | |  |  |
| David |  | 4 | 6 |  | 1 |  | |  |  |
| Thomas |  | 2 | 7 | 2 | 2 | 1 | |  | **Low** |
| Kylo |  | 2 | 6 | 2 | 2 | 2 | |  |  |
| Hakeem |  | 2 | 6 |  | ***6*** | 2 | |  |  |
| Zayn |  | 2 | 6 | 3 | ***7*** | 2 | |  |  |
| Mikael |  | 2 | 7 | 2 | ***6*** | 1 | |  |  |
| Zyaire |  | 2 | 6 | 2 | 1 | 2 | | **Yes** |  |

*Note*. Columns 1-6 show by how many classmates a student is named as a bully (column 1; “Who starts when are you victimized?”), a best friend (column 2; “Which classmates are your best friends?), liked (column 3”; “Which classmates do you like?), disliked (column 4”; “Which classmates do you dislike?”), or as popular (column 5; “Which classmates are popular?”), or leader (column 6; “Which classmates are good leaders?”). For example: Anna is named by three classmates as a bully and by 5 classmates as a friend. Columns 7-8 show worrisome levels of self-reported victimization and school safety and well-being (low-very low). Numbers in italic refer to low or high scores.

Table A.3.1 provides an example of the (network) feedback that teachers received in the KiVa+ condition. The information was derived from the online measurements for all students in the peer group. This table is taken from another study (Kaufman, Huitsing, Bloemberg, & Veenstra, 2019), and shows the classroom average number of nominations for each measure per student, and per student the aggregated number of received nominations (columns 1-6), and worrisome levels of self-reported victimization frequency and school safety and well-being (columns 7, 8). The table emphasizes the scores of the students who stand out because of their low or high scores: those who are nominated at least once as a bully or by no one as a friend, who are least liked (by <20% of the classmates) or most liked (by >35% of the classmates), and the students who are most rejected, most popular, most often considered a good leader, or considered as prosocial (by >20% of the classmates).

For example, the Table shows that one student, Sophie, reports to be systematically victimized, and combined with Sophie’s very low school well-being this information indicates that Sophie’s situation may be problematic. The network nominations reveal that she has no best friends and is only liked by two others. Sophie thus seems to have a marginalized position, which can make it more attractive for bullies to target her.

# Appendix 4: CONSORT 2010 Checklist

| Section/Topic | Item No | Checklist item | Reported in section |
| --- | --- | --- | --- |
| Title and abstract | | | |
|  | 1a | Identification as a randomised trial in the title | 🗹 |
|  | 1b | Structured summary of trial design, methods, results, and conclusions (for specific guidance see CONSORT for abstracts) | 🗹 |
| Introduction | | | |
| Background and objectives | 2a | Scientific background and explanation of rationale | 🗹 See introduction, specifically *The Present Study*. |
|  | 2b | Specific objectives or hypotheses | 🗹 See final paragraph of *Introduction* |
| Methods | | | |
| Trial design | 3a | Description of trial design (such as parallel, factorial) including allocation ratio | 🗹 See *Sampling and Design* |
|  | 3b | Important changes to methods after trial commencement (such as eligibility criteria), with reasons | Not applicable |
| Participants | 4a | Eligibility criteria for participants | 🗹 See *Sampling and Design* and *Limitations* for a discussion on representativeness |
|  | 4b | Settings and locations where the data were collected | 🗹 See *Sampling and Design* and *Data Collection and Participants*. |
| Interventions | 5 | The interventions for each group with sufficient details to allow replication, including how and when they were actually administered | 🗹 See Appendices 1, 2, and 3. |
| Outcomes | 6a | Completely defined pre-specified primary and secondary outcome measures, including how and when they were assessed | 🗹 See final paragraph of *Introduction* and see *Measures*. |
|  | 6b | Any changes to trial outcomes after the trial commenced, with reasons | 🗹 Not applicable, see pre-registering at trialregister.nl/trial/3903 |
| Sample size | 7a | How sample size was determined | 🗹 See Appendix 2.5 |
|  | 7b | When applicable, explanation of any interim analyses and stopping guidelines | Not applicable |
| Randomisation: |  |  |  |
| Sequence generation | 8a | Method used to generate the random allocation sequence | 🗹 See *Sampling and Design* and Appendix 2.6 |
|  | 8b | Type of randomisation; details of any restriction (such as blocking and block size) | 🗹 See *Sampling and Design* and Appendix 2.6 |
| Allocation concealment mechanism | 9 | Mechanism used to implement the random allocation sequence (such as sequentially numbered containers), describing any steps taken to conceal the sequence until interventions were assigned | 🗹 See *Sampling and Design* and Appendix 2.6 |
| Implementation | 10 | Who generated the random allocation sequence, who enrolled participants, and who assigned participants to interventions | 🗹 See Appendix 2 |
| Blinding | 11a | If done, who was blinded after assignment to interventions (for example, participants, care providers, those assessing outcomes) and how | 🗹 Blinding was not applied. Schools, trainers, and researchers were aware of the intervention status of schools. |
|  | 11b | If relevant, description of the similarity of interventions | Not applicable |
| Statistical methods | 12a | Statistical methods used to compare groups for primary and secondary outcomes | 🗹 See *Analytical Strategy* |
|  | 12b | Methods for additional analyses, such as subgroup analyses and adjusted analyses | 🗹 See *Analytical Strategy* (cross-level interactions of Intervention with *Boy* and *Grade*) |
| Results | | | |
| Participant flow (a diagram is strongly recommended) | 13a | For each group, the numbers of participants who were randomly assigned, received intended treatment, and were analysed for the primary outcome | 🗹 See *Figure 1* |
|  | 13b | For each group, losses and exclusions after randomisation, together with reasons | 🗹 See *Figure 1* |
| Recruitment | 14a | Dates defining the periods of recruitment and follow-up | 🗹 See *Method* and *Appendix 2* |
|  | 14b | Why the trial ended or was stopped | Not applicable |
| Baseline data | 15 | A table showing baseline demographic and clinical characteristics for each group | 🗹 See final paragraph of *Data Collection and Participants* for gender, age, and ethnicity, and Tables 1 and 2 (and Appendix 5) for baseline comparisons of the dependent variables |
| Numbers analysed | 16 | For each group, number of participants (denominator) included in each analysis and whether the analysis was by original assigned groups | 🗹 See *Figure 1* |
| Outcomes and estimation | 17a | For each primary and secondary outcome, results for each group, and the estimated effect size and its precision (such as 95% confidence interval) | 🗹 See Table 4 and Appendix 8 |
|  | 17b | For binary outcomes, presentation of both absolute and relative effect sizes is recommended | 🗹 See Table 4, Appendix 8, and the section *Logistic Regressions* for the relative risks |
| Ancillary analyses | 18 | Results of any other analyses performed, including subgroup analyses and adjusted analyses, distinguishing pre-specified from exploratory | 🗹 See *Results: Gender and Grade* and *KiVa+* |
| Harms | 19 | All important harms or unintended effects in each group (for specific guidance see CONSORT for harms) | 🗹 See *Discussion* |
| Discussion | | | |
| Limitations | 20 | Trial limitations, addressing sources of potential bias, imprecision, and, if relevant, multiplicity of analyses | 🗹 See *Discussion* (*Limitations, Strengths, and Future Directions*) |
| Generalisability | 21 | Generalisability (external validity, applicability) of the trial findings | 🗹 See *2^nd^ paragraph of* (*Limitations, Strengths, and Future Directions* (starting with “The sample was not representative for the Dutch population”) |
| Interpretation | 22 | Interpretation consistent with results, balancing benefits and harms, and considering other relevant evidence | 🗹 See 2^nd^ paragraph of “Unique Societal Circumstances during the RCT |
| Other information | | |  |
| Registration | 23 | Registration number and name of trial registry | 🗹 trialregister.nl/trial/3903 |
| Protocol | 24 | Where the full trial protocol can be accessed, if available | Not available |
| Funding | 25 | Sources of funding and other support (such as supply of drugs), role of funders | 🗹 |

# Appendix 5: Absolute and Relative Numbers of Victims and Bullies (Maximum Scores)

**Table A5.1**. Self-Reported **Victimization (Maximum on Ten Specific Items)** in Control and Intervention Schools in the Focus Cohort (Grades 3-4)

| **Intervention** | T1 | | |  | T2 | |  | T3 | |  | T4 | |  | T5 | |
| --- | --- | --- | --- | --- | --- | --- | --- | --- | --- | --- | --- | --- | --- | --- | --- |
|  | N | % | |  | N | % |  | N | % |  | N | % |  | N | % |
|  | 2987 |  | |  | 3181 |  |  | 3133 |  |  | 2990 |  |  | 2933 |  |
| Not at all | 747 | 25.0% | |  | 1058 | 33.3% |  | 1419 | 45.3% |  | 1659 | 55.5% |  | 1784 | 60.8% |
| % change^1^ |  |  | |  |  |  |  |  | **+81.1%** |  |  |  |  |  | **+143.2%** |
| Once/twice | 763 | 25.5% | |  | 885 | 27.8% |  | 788 | 25.2% |  | 696 | 23.3% |  | 632 | 21.5% |
| % change^1^ |  |  | |  |  |  |  |  | **-1.5%** |  |  |  |  |  | **-15.6%** |
| Monthly | 444 | 14.9% | |  | 437 | 13.7% |  | 354 | 11.3% |  | 274 | 9.2% |  | 239 | 8.1% |
| % change^1^ |  |  | |  |  |  |  |  | **-24.0%** |  |  |  |  |  | **-45.2%** |
| Weekly | 287 | 9.6% | |  | 228 | 7.2% |  | 218 | 7.0% |  | 139 | 4.6% |  | 116 | 4.0% |
| % change^1^ |  |  | |  |  |  |  |  | **-27.6%** |  |  |  |  |  | **-58.8%** |
| Daily | 746 | 25.0% | |  | 573 | 18.0% |  | 354 | 11.3% |  | 222 | 7.4% |  | 162 | 5.5% |
| % change^1^ |  |  | |  |  |  |  |  | **-54.8%** |  |  |  |  |  | **-77.9%** |
| Total victims^2^ | 1477 | 49.4% | |  | 1238 | 38.9% |  | 926 | 29.6% |  | 635 | 21.2% |  | 517 | 17.6% |
| % change^1^ |  |  | |  |  |  |  |  | **-40.2%** |  |  |  |  |  | **-64.4%** |
|  |  |  | |  |  |  |  |  |  |  |  |  |  |  |  |
| **Control** | T1 | | |  | T2 | |  | T3 | |  | T4 | |  | T5 | |
|  | N | | % |  | N | % |  | N | % |  | N | % |  | N | % |
|  | 1288 | |  |  | 1346 |  |  | 1326 |  |  | 1307 |  |  | 1306 |  |
| Not at all | 344 | | 26.7% |  | 463 | 34.4% |  | 522 | 39.4% |  | 641 | 49.0% |  | 680 | 52.1% |
| % change^1^ |  | |  |  |  |  |  |  | **+47.4%** |  |  |  |  |  | **+94.9%** |
| Once/twice | 339 | | 26.3% |  | 354 | 26.3% |  | 348 | 26.2% |  | 334 | 25.6% |  | 318 | 24.3% |
| % change^1^ |  | |  |  |  |  |  |  | **-0.3%** |  |  |  |  |  | **-7.5%** |
| Monthly | 206 | | 16.0% |  | 178 | 13.2% |  | 157 | 11.8% |  | 143 | 10.9% |  | 140 | 10.7% |
| % change^1^ |  | |  |  |  |  |  |  | **-26.0%** |  |  |  |  |  | **-33.0%** |
| Weekly | 115 | | 8.9% |  | 96 | 7.1% |  | 120 | 9.0% |  | 74 | 5.7% |  | 70 | 5.4% |
| % change^1^ |  | |  |  |  |  |  |  | **1.4%** |  |  |  |  |  | **-40.0%** |
| Daily | 284 | | 22.0% |  | 255 | 18.9% |  | 179 | 13.5% |  | 115 | 8.8% |  | 98 | 7.5% |
| % change^1^ |  | |  |  |  |  |  |  | **-38.8%** |  |  |  |  |  | **-66.0%** |
| Total victims^2^ | 605 | | 47.0% |  | 529 | 39.3% |  | 456 | 34.4% |  | 332 | 25.4% |  | 308 | 23.6% |
| % change^1^ |  | |  |  |  |  |  |  | **-26.8%** |  |  |  |  |  | **-49.8%** |

*Notes.*

^1^ The percentages of change are calculated relative to the T1 prevalence.

^2^ The sum of victims who are victimized monthly, daily, or weekly.

**Table A5.2**. Self-Reported **Bullying** **(Maximum on Ten Specific Items)** in Control and Intervention Schools in the Focus Cohort (Grades 3-4)

| **Intervention** | T1 | |  | T2 | |  | T3 | |  | T4 | |  | T5 | |
| --- | --- | --- | --- | --- | --- | --- | --- | --- | --- | --- | --- | --- | --- | --- |
|  | N | % |  | N | % |  | N | % |  | N | % |  | N | % |
|  | 2983 |  |  | 3178 |  |  | 3132 |  |  | 2990 |  |  | 2933 |  |
| Not at all | 1696 | 56.9% |  | 2083 | 65.5% |  | 2338 | 74.6% |  | 2357 | 78.8% |  | 2405 | 82.0% |
| % change^1^ |  |  |  |  |  |  |  | **+31.3%** |  |  |  |  |  | **+44.2%** |
| Once/twice | 785 | 26.3% |  | 731 | 23.0% |  | 566 | 18.1% |  | 474 | 15.9% |  | 424 | 14.5% |
| % change^1^ |  |  |  |  |  |  |  | **-31.3%** |  |  |  |  |  | **-45.1%** |
| Monthly | 191 | 6.4% |  | 147 | 4.6% |  | 87 | 2.8% |  | 81 | 2.7% |  | 55 | 1.9% |
| % change^1^ |  |  |  |  |  |  |  | **-56.6%** |  |  |  |  |  | **-70.7%** |
| Weekly | 114 | 3.8% |  | 62 | 2.0% |  | 48 | 1.5% |  | 28 | 0.9% |  | 20 | 0.7% |
| % change^1^ |  |  |  |  |  |  |  | **-59.9%** |  |  |  |  |  | **-82.2%** |
| Daily | 197 | 6.6% |  | 155 | 4.9% |  | 93 | 3.0% |  | 50 | 1.7% |  | 29 | 1.0% |
| % change^1^ |  |  |  |  |  |  |  | **-55.0%** |  |  |  |  |  | **-85.0%** |
| Total bullies^2^ | 502 | 16.8% |  | 364 | 11.5% |  | 228 | 7.3% |  | 159 | 5.3% |  | 104 | 3.5% |
| % change^1^ |  |  |  |  |  |  |  | **-56.7%** |  |  |  |  |  | **-78.9%** |
|  | | | | | | | | | | | | | | |
| **Control** | T1 | |  | T2 | |  | T3 | |  | T4 | |  | T5 | |
|  | N | % |  | N | % |  | N | % |  | N | % |  | N | % |
|  | 1284 |  |  | 1344 |  |  | 1324 |  |  | 1303 |  |  | 1306 |  |
| Not at all | 765 | 59.6% |  | 913 | 67.9% |  | 929 | 70.2% |  | 973 | 74.7% |  | 1004 | 76.9% |
| % change^1^ |  |  |  |  |  |  |  | **+17.8%** |  |  |  |  |  | **+29.0%** |
| Once/twice | 324 | 25.2% |  | 295 | 21.9% |  | 288 | 21.8% |  | 253 | 19.4% |  | 242 | 18.5% |
| % change^1^ |  |  |  |  |  |  |  | **-13.8%** |  |  |  |  |  | **-26.6%** |
| Monthly | 84 | 6.5% |  | 49 | 3.6% |  | 52 | 3.9% |  | 41 | 3.1% |  | 37 | 2.8% |
| % change^1^ |  |  |  |  |  |  |  | **-40.0%** |  |  |  |  |  | **-56.7%** |
| Weekly | 29 | 2.3% |  | 18 | 1.3% |  | 20 | 1.5% |  | 15 | 1.2% |  | 10 | 0.8% |
| % change^1^ |  |  |  |  |  |  |  | **-33.1%** |  |  |  |  |  | **-66.1%** |
| Daily | 82 | 6.4% |  | 69 | 5.1% |  | 35 | 2.6% |  | 21 | 1.6% |  | 13 | 1.0% |
| % change^1^ |  |  |  |  |  |  |  | **-58.6%** |  |  |  |  |  | **-84.4%** |
| Total bullies^2^ | 195 | 15.2% |  | 136 | 10.1% |  | 107 | 8.1% |  | 77 | 5.9% |  | 60 | 4.6% |
| % change^1^ |  |  |  |  |  |  |  | **-46.8%** |  |  |  |  |  | **-69.7%** |

*Notes.*

^1^ The percentages of change are calculated relative to the T1 prevalence.

^2^ The sum of bullies who bully monthly, daily, or weekly.

# Appendix 6: Multinomial Logistic Regressions for KiVa and KiVa+

**Table A6.1** Multinomial Logistic Regressions to Estimate the Effects of **KiVa and KiVa+** on **Self-reported Victimization**

|  | **KiVa vs. KiVa+ Global victimization** | | |  | **KiVa vs. KiVa+ Victimization Max** | | |
| --- | --- | --- | --- | --- | --- | --- | --- |
|  | Est. | *SE* | *OR* |  | Est. | *SE* | *OR* |
| **Intercept** |  |  |  |  |  |  |  |
| *2: Occasionally* | 0.80** | (0.10) | 2.23 |  | 1.64** | (0.10) | 5.18 |
| *3: Monthly* | -0.91** | (0.10) | 0.40 |  | 0.00 | (0.10) | 1.00 |
| *4: Weekly* | -1.59** | (0.10) | 0.20 |  | -0.93** | (0.10) | 0.40 |
| *5: Daily* | -2.25** | (0.10) | 0.11 |  | -1.60** | (0.10) | 0.20 |
| **KiVa+ (KiVa=ref)** | -0.03 | (0.14) | 0.97 |  | 0.01 | (0.14) | 0.99 |
| **Change by wave 2** |  |  |  |  |  |  |  |
| T2 | -0.55** | (0.07) | 0.58 |  | -0.59** | (0.07) | 0.55 |
| KiVa+ **×** T2 | 0.07 | (0.10) | 1.07 |  | 0.01 | (0.10) | 1.01 |
| **Change by wave 3** |  |  |  |  |  |  |  |
| T3 | -1.15** | (0.08) | 0.32 |  | -1.29** | (0.07) | 0.27 |
| KiVa+ **×** T3 | 0.14 | (0.11) | 1.15 |  | 0.01 | (0.10) | 1.01 |
| **Change by wave 4** |  |  |  |  |  |  |  |
| T4 | -1.75** | (0.08) | 0.17 |  | -1.94** | (0.08) | 0.14 |
| KiVa+ **×** T4 | 0.18 | (0.11) | 1.20 |  | 0.14 | (0.11) | 1.16 |
| **Change by wave 5** |  |  |  |  |  |  |  |
| T5 | -1.91** | (0.09) | 0.15 |  | -2.22** | (0.08) | 0.11 |
| KiVa+ **×** T5 | 0.16 | (0.12) | 1.17 |  | 0.08 | (0.11) | 1.08 |
| **Variance components** |  |  |  |  |  |  |  |
| School level | 0.09 | (0.04) |  |  | 0.06 | (0.04) |  |
| Classroom level T1 | 0.08 | (0.06) |  |  | 0.09 | (0.07) |  |
| Classroom level T3 | 0.07 | (0.06) |  |  | 0.06 | (0.06) |  |
| Classroom level T5 | 0.02 | (0.03) |  |  | 0.04 | (0.04) |  |
| Student level | 2.66 | (0.12) |  |  | 2.72 | (0.12) |  |

*Note.* ** *p* < .01.

**Table A6.2** Multinomial Logistic Regressions to Estimate the Effects of **KiVa and KiVa+** on **Self-reported Bullying**

|  | **KiVa vs. KiVa+ Global bullying** | | | **KiVa vs. KiVa+ Bullying Max** | | | |
| --- | --- | --- | --- | --- | --- | --- | --- |
|  | Est. | *SE* | *OR* | Est. | *SE* | *OR* |  |
| **Intercept** |  |  |  |  |  |  |  |
| *2: Occasionally* | -0.79** | (0.13) | 0.46 | -0.31 | (0.14) | 0.73 |  |
| *3: Monthly* | -2.86** | (0.14) | 0.06 | -2.34** | (0.14) | 0.10 |  |
| *4: Weekly* | -3.66** | (0.14) | 0.03 | -3.09** | (0.15) | 0.05 |  |
| *5: Daily* | -4.26** | (0.15) | 0.01 | -3.62** | (0.15) | 0.03 |  |
| **KiVa+ (KiVa=ref)** | -0.05 | (0.19) | 0.96 | 0.02 | (0.20) | 1.03 |  |
| **Change by wave 2** |  |  |  |  |  |  |  |
| T2 | -0.50** | (0.09) | 0.61 | -0.58** | (0.08) | 0.56 |  |
| KiVa+ **×** T2 | 0.00 | (0.12) | 1.00 | 0.01 | (0.12) | 1.01 |  |
| **Change by wave 3** |  |  |  |  |  |  |  |
| T3 | -0.83** | (0.09) | 0.44 | -1.13** | (0.09) | 0.32 |  |
| KiVa+ **×** T3 | -0.04 | (0.13) | 0.96 | -0.16 | (0.13) | 0.85 |  |
| **Change by wave 4** |  |  |  |  |  |  |  |
| T4 | -1.28** | (0.10) | 0.28 | -1.60** | (0.10) | 0.20 |  |
| KiVa+ **×** T4 | 0.16 | (0.14) | 1.18 | 0.10 | (0.13) | 1.10 |  |
| **Change by wave 5** |  |  |  |  |  |  |  |
| T5 | -1.48** | (0.10) | 0.23 | -1.81** | (0.10) | 0.16 |  |
| KiVa+ **×** T5 | 0.06 | (0.14) | 1.06 | -0.04 | (0.14) | 0.96 |  |
| **Variance components** |  |  |  |  |  |  |  |
| School level | 0.26 | (0.08) |  | 0.28 | (0.09) |  |  |
| Classroom level T1 | 0.13 | (0.07) |  | 0.20 | (0.09) |  |  |
| Classroom level T3 | 0.07 | (0.06) |  | 0.06 | (0.07) |  |  |
| Classroom level T5 | 0.04 | (0.05) |  | 0.04 | (0.05) |  |  |
| Student level | 2.27 | (0.13) |  | 2.54 | (0.14) |  |  |

*Note.* ** *p* < .01.

# Appendix 7: Logistic Regressions

**Table A7.1: Logistic Regressions** to Estimate the Effects of the Intervention on **Self-Reported Victimization and Bullying**

|  | **Victimization  (Global)** | | | |  | **Victimization (Maximum)** | | | |  | **Bullying (Global)** | | | |  | **Bullying (Maximum)** | | | |
| --- | --- | --- | --- | --- | --- | --- | --- | --- | --- | --- | --- | --- | --- | --- | --- | --- | --- | --- | --- |
|  | Est. | SE | OR | *p* |  | Est. | SE | OR | *p* |  | Est. | SE | OR | *p* |  | Est. | SE | OR | *p* |
| **Intercept** | -0.89 | (0.15) | 0.41 | <.001 |  | 0.08 | (0.15) | 1.08 | .589 |  | -3.28 | (0.21) | 0.04 | <.001 |  | -2.52 | (0.21) | 0.08 | <.001 |
| **KiVa** | 0.14 | (0.18) | 1.15 | .450 |  | 0.13 | (0.18) | 1.13 | .484 |  | 0.05 | (0.24) | 1.05 | .839 |  | 0.01 | (0.24) | 1.01 | .960 |
| **Boy** | 0.01 | (0.13) | 1.01 | .922 |  | -0.05 | (0.12) | 0.95 | .703 |  | 0.82 | (0.16) | 2.26 | <.001 |  | 0.77 | (0.15) | 2.17 | <.001 |
| **Grade 4** | -0.31 | (0.15) | 0.73 | .034 |  | -0.38 | (0.14) | 0.69 | .007 |  | -0.15 | (0.18) | 0.86 | .387 |  | -0.47 | (0.17) | 0.62 | .006 |
| **KiVa × Grade 4** | -0.13 | (0.17) | 0.88 | .446 |  | -0.12 | (0.17) | 0.88 | .465 |  | -0.24 | (0.21) | 0.79 | .261 |  | -0.08 | (0.21) | 0.92 | .687 |
| **KiVa × Boy** | 0.08 | (0.15) | 1.09 | .584 |  | 0.13 | (0.15) | 1.13 | .389 |  | 0.10 | (0.19) | 1.11 | .587 |  | 0.14 | (0.18) | 1.15 | .429 |
| **Change wave 2** |  |  |  |  |  |  |  |  |  |  |  |  |  |  |  |  |  |  |  |
| T2 | -0.40 | (0.11) | 0.67 | <.001 |  | -0.50 | (0.10) | 0.61 | <.001 |  | -0.28 | (0.15) | 0.76 | .071 |  | -0.63 | (0.14) | 0.53 | <.001 |
| KiVa **×** T2 | -0.19 | (0.13) | 0.82 | .127 |  | -0.16 | (0.12) | 0.85 | .238 |  | -0.20 | (0.18) | 0.82 | .269 |  | 0.01 | (0.16) | 1.01 | .947 |
| **Change wave 3** |  |  |  |  |  |  |  |  |  |  |  |  |  |  |  |  |  |  |  |
| T3 | -0.62 | (0.11) | 0.54 | <.001 |  | -0.82 | (0.10) | 0.44 | <.001 |  | -0.58 | (0.16) | 0.56 | <.001 |  | -0.93 | (0.15) | 0.39 | <.001 |
| KiVa **×** T3 | -0.54 | (0.13) | 0.58 | <.001 |  | -0.48 | (0.12) | 0.62 | <.001 |  | -0.22 | (0.19) | 0.80 | .256 |  | -0.37 | (0.18) | 0.69 | .037 |
| **Change wave 4** |  |  |  |  |  |  |  |  |  |  |  |  |  |  |  |  |  |  |  |
| T4 | -1.24 | (0.12) | 0.29 | <.001 |  | -1.48 | (0.11) | 0.23 | <.001 |  | -1.15 | (0.19) | 0.32 | <.001 |  | -1.33 | (0.16) | 0.26 | <.001 |
| KiVa **×** T4 | -0.57 | (0.14) | 0.57 | <.001 |  | -0.44 | (0.13) | 0.64 | <.001 |  | -0.13 | (0.22) | 0.88 | .555 |  | -0.35 | (0.20) | 0.71 | .077 |
| **Change wave 5** |  |  |  |  |  |  |  |  |  |  |  |  |  |  |  |  |  |  |  |
| T5 | -1.43 | (0.12) | 0.24 | <.001 |  | -1.64 | (0.11) | 0.19 | <.001 |  | -1.52 | (0.20) | 0.22 | <.001 |  | -1.68 | (0.18) | 0.19 | <.001 |
| KiVa **×** T5 | -0.47 | (0.15) | 0.62 | <.001 |  | -0.61 | (0.13) | 0.54 | <.001 |  | -0.23 | (0.25) | 0.79 | .348 |  | -0.50 | (0.22) | 0.61 | .021 |
| **Variance** |  |  |  |  |  |  |  |  |  |  |  |  |  |  |  |  |  |  |  |
| School level | 0.10 | (0.04) |  |  |  | 0.11 | (0.04) |  |  |  | 0.22 | (0.07) |  |  |  | 0.39 | (0.10) |  |  |
| Classroom level T1 | 0.05 | (0.04) |  |  |  | 0.06 | (0.05) |  |  |  | 0.06 | (0.06) |  |  |  | 0.12 | (0.07) |  |  |
| Classroom level T3 | 0.05 | (0.04) |  |  |  | 0.03 | (0.03) |  |  |  | 0.06 | (0.07) |  |  |  | 0.03 | (0.04) |  |  |
| Classroom level T5 | 0.02 | (0.03) |  |  |  | 0.03 | (0.04) |  |  |  | 0.06 | (0.06) |  |  |  | 0.03 | (0.04) |  |  |
| Student level | 3.12 | (0.16) |  |  |  | 3.17 | (0.15) |  |  |  | 2.33 | (0.20) |  |  |  | 2.53 | (0.19) |  |  |

**Table A7.2** Logistic Regressions to Estimate the Effects of **KiVa and KiVa+** on **Self-reported Victimization**

|  | **KiVa vs. KiVa+ Global victimization** | |  |  | **KiVa vs. KiVa+ Victimization Max** | | |
| --- | --- | --- | --- | --- | --- | --- | --- |
|  | Est. | *SE* | *OR* |  | Est. | *SE* | *OR* |
| **Intercept** | -0.86 | (0.11) | 0.42 |  | 0.06 | (0.11) | 1.06 |
| **KiVa+ (KiVa=ref)** | -0.09 | (0.15) | 0.92 |  | -0.13 | (0.16) | 0.88 |
| **Change by wave 2** |  |  |  |  |  |  |  |
| T2 | -0.61** | (0.10) | 0.54 |  | -0.72** | (0.09) | 0.49 |
| KiVa+ **×** T2 | 0.04 | (0.14) | 1.04 |  | 0.14 | (0.13) | 1.15 |
| **Change by wave 3** |  |  |  |  |  |  |  |
| T3 | -1.24** | (0.11) | 0.29 |  | -1.32** | (0.10) | 0.27 |
| KiVa+ **×** T3 | 0.19 | (0.15) | 1.21 |  | 0.07 | (0.14) | 1.07 |
| **Change by wave 4** |  |  |  |  |  |  |  |
| T4 | -1.91** | (0.12) | 0.15 |  | -2.02** | (0.11) | 0.13 |
| KiVa+ **×** T4 | 0.23 | (0.16) | 1.26 |  | 0.22 | (0.14) | 1.25 |
| **Change by wave 5** |  |  |  |  |  |  |  |
| T5 | -2.10** | (0.12) | 0.12 |  | -2.40** | (0.11) | 0.09 |
| KiVa+ **×** T5 | 0.40 | (0.17) | 1.49 |  | 0.33 | (0.15) | 1.39 |
| **Variance components** |  |  |  |  |  |  |  |
| School level | 0.06 | (0.05) |  |  | 0.13 | (0.05) |  |
| Classroom level T1 | 0.06 | (0.05) |  |  | 0.07 | (0.05) |  |
| Classroom level T3 | 0.04 | (0.04) |  |  | 0.05 | (0.05) |  |
| Classroom level T5 | 0.04 | (0.04) |  |  | 0.04 | (0.04) |  |
| Student level | 2.98 | (0.19) |  |  | 2.98 | (0.17) |  |

*Note.* ** *p* < .01.

**Table A7.3** Logistic Regressions to Estimate the Effects of **KiVa and KiVa+** on **Self-reported Bullying**

|  | **KiVa vs. KiVa+ Global bullying** | | |  | **KiVa vs. KiVa+ Bullying Max** | | |
| --- | --- | --- | --- | --- | --- | --- | --- |
|  | Est. | *SE* | *OR* |  | Est. | *SE* | *OR* |
| **Intercept** | -3.01 | (0.17) | 0.05 |  | -2.40 | (0.18) | 0.09 |
| **KiVa+ (KiVa=ref)** | 0.08 | (0.22) | 1.08 |  | 0.18 | (0.24) | 1.19 |
| **Change by wave 2** |  |  |  |  |  |  |  |
| T2 | -0.42** | (0.14) | 0.66 |  | -0.47** | (0.12) | 0.63 |
| KiVa+ **×** T2 | -0.14 | (0.20) | 0.87 |  | -0.31 | (0.18) | 0.73 |
| **Change by wave 3** |  |  |  |  |  |  |  |
| T3 | -0.83** | (0.15) | 0.44 |  | -1.16** | (0.14) | 0.31 |
| KiVa+ **×** T3 | 0.05 | (0.21) | 1.05 |  | -0.29 | (0.20) | 0.75 |
| **Change by wave 4** |  |  |  |  |  |  |  |
| T4 | -1.34** | (0.18) | 0.26 |  | -1.74** | (0.16) | 0.18 |
| KiVa+ **×** T4 | 0.10 | (0.24) | 1.11 |  | 0.44 | (0.22) | 1.11 |
| **Change by wave 5** |  |  |  |  |  |  |  |
| T5 | -1.86** | (0.20) | 0.15 |  | -2.10** | (0.18) | 0.12 |
| KiVa+ **×** T5 | 0.20 | (0.28) | 1.22 |  | -0.14 | (0.26) | 0.87 |
| **Variance components** |  |  |  |  |  |  |  |
| School level | 0.28 | (0.10) |  |  | 0.49 | (0.14) |  |
| Classroom level T1^1^ | 0.04 | (0.08) |  |  | 0.07 | (0.08) |  |
| Classroom level T3 | 0.13 | (0.10) |  |  | 0.08 | (0.09) |  |
| Classroom level T5 | 0.05 | (0.08) |  |  | 0.12 | (0.10) |  |
| Student level | 2.51 | (0.26) |  |  | 2.64 | (0.24) |  |

*Note.* ** *p* < .01.

^1^ ESS = 143 for classroom level variance for global bullying.

#

#

# Appendix 8: Overview of ORs and CIs for the KiVa and KiVa+ Effects

**Table A8.1** Overview of Odds Ratio’s and Confidence Intervals for the **KiVa Effects Compared to KiVa+ Effects**

|  | **Frequency  (Multinomial Logistic Regressions)** | | | |  | **Occurrence**  **(Binomial Logistic Regressions)** | | | |  |
| --- | --- | --- | --- | --- | --- | --- | --- | --- | --- | --- |
|  | OR | *p* | 95% CI | Inverse  OR |  | OR | *p* | 95% CI | Inverse OR | |
| **Victimization** |  |  |  |  |  |  |  |  |  | |
| Global, T3^1^ | 1.12 | .433 | 0.85 – 1.47 | 0.90 |  | 1.11 | .523 | 0.81 –1.53 | 0.90 | |
| Global, T5^2^ | 1.16 | .322 | 0.86 – 1.56 | 0.86 |  | 1.37 | .084 | 0.96 – 1.96 | 0.73 | |
| Maximum, T3 | 1.01 | .960 | 0.76 – 1.34 | 0.99 |  | 0.94 | .705 | 0.69 – 1.29 | 1.06 | |
| Maximum, T5 | 1.14 | .379 | 0.85 – 1.53 | 0.88 |  | 1.22 | .257 | 0.86 – 1.73 | 0.82 | |
| **Bullying** |  |  |  |  |  |  |  |  |  | |
| Global, T3 | 0.92 | .640 | 0.63 – 1.35 | 1.09 |  | 1.14 | .597 | 0.71 – 1.84 | 0.88 | |
| Global, T5 | 1.01 | .966 | 0.68 – 1.52 | 0.99 |  | 1.32 | .355 | 0.73 – 2.40 | 0.76 | |
| Maximum, T3 | 0.87 | .492 | 0.58 – 1.30 | 1.15 |  | 0.89 | .667 | 0.54 – 1.51 | 1.12 | |
| Maximum, T5 | 0.99 | .950 | 0.65 – 1.50 | 1.01 |  | 1.04 | .904 | 0.57 – 1.91 | 0.96 | |

*Note.* ^1^ T3 is one year after implementing the intervention. ^2^ T5 is two years after implementing the intervention.

# References

Garandeau, C. F., Poskiparta, E., & Salmivalli, C. (2014). Tackling acute cases of school bullying in the KiVa anti-bulying program: A comparison of two approaches. *Journal of Abnormal Child Psychology*, *42*, 981–991.

Hoffman, T. C., Glasziou, P. P., Boutron, I., Milne, R., Perera, R., Moher, D., … Michie, S. (2014). Better reporting of interventions: template for intervention description and replication (TIDieR) checklist. *BMJ*, *348*.

Kärnä, A., Voeten, M., Little, T. D., Poskiparta, E., Kaljonen, A., & Salmivalli, C. (2011). A large-scale evaluation of the KiVa antibullying program: Grades 4-6. *Child Development*, *82*, 311–330.

Kaufman, T. M. L., Huitsing, G., Bloemberg, R., & Veenstra, R. (2019). The Systematic Application of Network Diagnostics to Monitor and Tackle Bullying and Victimization in Schools. *Under Review*.

Olweus, D. (1996). *The revised Olweus bully/victim questionnaire*. Bergen, Norway: Research Center for Health Promotion (HEMIL Center), University of Bergen.

Rigby, K. (2014). How teachers address cases of bullying in schools: A comparison of five reactive approaches. *Educational Psychology in Practice*, *30*, 409–419.

Robinson, G., & Maines, B. (2008). *Bullying: A complete guide to the support group method*. London: Sage.

Saarento, S., Boulton, A. J., & Salmivalli, C. (2015). Reducing bullying and victimization: Student- and classroom-level mechanisms of change. *Journal of Abnormal Child Psychology*, *43*, 61–76.

Spybrook, J., Bloom, H., Congdon, R., Hill, C., Martinez, A., & Raudenbush, S. (2011). Optimal Design plus empirical evidence: Documentation for the “Optimal Design” software.

Van der Ploeg, R., Steglich, C., & Veenstra, R. (2016). The Support Group Approach in the Dutch KiVa anti-bullying programme: Effects on victimisation, defending, and well-being at school. *Educational Research*, *3*, 221–236.

Young, S. (1998). The Support Group Approach to bullying in schools. *Educational Psychology in Practice*, *14*, 32–39.
